# Internet-delivered Cognitive Behavioral Therapy for insomnia in youth with autism spectrum disorder: A pilot study

**DOI:** 10.1016/j.invent.2022.100548

**Published:** 2022-05-23

**Authors:** Lisa Georén, Markus Jansson-Fröjmark, Lisa Nordenstam, Gerhard Andersson, Nora Choque Olsson

**Affiliations:** a Stockholm Health Care Services, Stockholm County Council, Stockholm, Sweden; b Center for Psychiatry Research, Department of Clinical Neuroscience, Karolinska Institutet and Stockholm Health Care Services, Stockholm County Council, Stockholm, Sweden; c Department of Behavioural Sciences and Learning, Department of Biomedical and Clinical Sciences, Linköping University, Linköping, Sweden; d Department of Psychology, Stockholm University, Stockholm, Sweden

**Keywords:** Autism spectrum disorder, Evidence-based intervention, Internet-delivered, Psychiatry, Sleep disorders, Insomnia

## Abstract

Adolescents with ASD often suffer from sleep disorders affecting their development and quality of life. Research concerning psychological treatment of insomnia in this population is scarce. The objective of this pilot study was to examine the feasibility of internet-delivered CBT for insomnia (iCBT-I) and the participants' experiences after completing the treatment. Both quantitative and qualitative methods were used. Six adolescents with ASD and insomnia, aged 13 to 17, participated in the study. The results of the qualitative investigation showed general satisfaction with the iCBT-I. The participants experienced both better sleep and insights into their sleep patterns. Thematic analysis revealed five themes: *experience of the structure of the treatment*, *treatment content*, *experienced outcomes*, *experienced difficulties*, and *suggested improvements*. The results indicate the need for additional support for some participants and suggest distinct recommendations for further studies. The quantitative investigation showed large reductions in insomnia symptoms indicating the feasibility of the treatment in this population. The findings suggest promising results, but more studies are needed to define the efficacy of iCBT-I for adolescents with ASD.

## Highlights

• Internet delivered CBT for insomnia (iCBT-I) is feasible in youths with ASD.
• Findings suggest large reductions in insomnia after completing the iCBT-I.
• General satisfaction with iCBT-I among youths with ASD.

## 1 Introduction

The prevalence of children and adolescents with autism spectrum disorder (ASD) experiencing sleep problems is markedly higher than in typically developing (TD) children and adolescents (between 40% to 80% in ASD and up to 40% in TD) (Mayes and Calhoun, 2009; Souders et al., 2009; Cortesi et al., 2010). Studies have shown that the sleep problems in TD children and adolescents often lessen with age (Mayes and Calhoun, 2009; Hodge et al., 2013), while sleep problems are persistent in children and adolescents with ASD (Corkum et al., 2014; McCrae et al., 2020a, McCrae et al., 2020b). Sleep disturbance seems to increase with autism severity (Mayes and Calhoun, 2009). A large-scale study with a sample of 1859 individuals with ASD (aged 3–18) found that younger children (<7 years of age) had more prominent issues with sleep anxiety, bedtime resistance, night awakenings, and parasomnias, whereas for adolescents (≥11 years) problems related to sleep onset, sleep duration, and daytime sleepiness (Goldman et al., 2012), emphasizing the need for clinicians to address sleep problems not only in children with ASD but throughout the age span (Goldman et al., 2012). Children with ASD and sleep problems have been found to have other symptoms and behavioral problems compared to those without (Park et al., 2012) and there is a consistent negative relationship between sleep disturbance and Health-Related Quality of Life (HRQoL) with greater sleep problems being associated with a lower HRQoL (Delahaye et al., 2014). Sleep problems also increase parental stress and negatively impact parental sleep, family quality of life, and day-to-day life (Hoffman and Smits, 2008; Hodge et al., 2013). These findings suggest that treatments for sleep disturbances in children and adolescents with ASD are needed (Delahaye et al., 2014).

Although behavioral sleep interventions are recommended as the initial approach to reduce insomnia in adults (Jansson-Frojmark and Norell-Clarke, 2016) few studies focusing on treatment for sleep problems for children with ASD have been conducted (Malow et al., 2006; Mullane and Corkum, 2006; Corkum et al., 2018). Earlier research on interventions examined in a systematic review revealed that interventions have been primarily directed to parents with younger children (<12 years) with a large focus on behavioral interventions, of which two was found to meet the criteria for possibly efficacious interventions according to the Chambless criteria for treatment efficacy; standard extinction and schedules awakenings, but more reaserach is needed to properly evaluate other interventions (Vriend et al., 2011). However, studies on behavioral interventions for adolescents with ASD and sleep problems have not been conducted. The purpose of the current pilot study was to evaluate the feasibility of behavioral treatment for adolescents with ASD (aged 13–18 years old) as a first step of a large evaluation of the treatment.

Cognitive-behavioral therapy for insomnia (CBT-I) is considered an evidence-based treatment for insomnia in adults (Bastien et al., 2004; Jansson-Frojmark and Norell-Clarke, 2016) and a study in primary school-aged children has shown significant improvement in sleep latency, waking after sleep onset, and sleep efficiency after CBT-I treatment (Shatkin JP, 2015). A recent review suggested that CBT-I for adolescents with insomnia can be adapted to an internet delivered format (Åslund et al., 2018) and studies on internet-delivered treatment for insomnia for children are being performed, such as an ongoing study by Corkum et al. (2018) for children (1–10 year) (Corkum et al., 2018). A study focusing on barriers of internet-delivered intervention for children and adolescents have also been published showing the importance of qualitative feedback on the interventions, describing what users expect from e-health programs in terms of user experience and why minimizing the need for outside support is important; directing the treatment to the parents (Speth et al., 2015). Although previous studies suggest a positive effect of CBT-I in adolescents, studies of iCBT-I in adolescents with ASD are needed (Hepburn et al., 2016). A recent study of telehealth delivery of CBT-I in children with ASD (6–12 years) has been published revealing promising results (McCrae et al., 2020a, McCrae et al., 2020b), but studies focusing on effectiveness of Internet-delivered CBT for insomnia (iCBT-I) for adolescents (aged 13–18) with ASD have not been conducted. This pilot study focuses on the experiences and views of adolescents with ASD about the iCBT-I after completing the treatment in order to improve the feasibility of the treatment to adolescents with ASD prior to a randomized controlled study.

The aims of this pilot study were to investigate the experiences of adolescents with ASD about iCBT-I after completing the treatment and the feasibility of the treatment. The questions addressed were: (1) How do adolescents with ASD and insomnia experience the content and structure of the treatment? (2) Did the adolescents with ASD participating in the iCBT-I face any particular difficulties that interfered with the treatment? (3) What improvements are needed to enhance the adaptation of iCBT-I for adolescents with ASD with sleep problems? (4) Is iCBT-I feasible in adolescents with ASD and insomnia? (5) What is the preliminary effect of iCBT-I on insomnia symptoms? The hypothesis based on the two latter questions are: (i) The iCBT-I is feasible in adolescents with ASD and (ii) the preliminary results of the treatment reduce insomnia symtoms in adolescents with ASD.

## 2 Material and methods

### 2.1 Design

A mixed-method approach that involved both qualitative evaluation and quantitative evaluation was applied (Subedi, 2016). The included participants were interviewed by using a semi-structured interview at post-treatment. The quantitative evaluation was based on self-report measures and sleep diaries at pre-treatment, mid-treatment, post-treatment, and two follow-ups. All participants and their parents provided written consent. The research was approved by the local ethics committee in Stockholm, Sweden (2017/1399-31). The pilot study was implemented between autumn 2018 and spring 2020.

### 2.2 Sample

In total, six adolescents with ASD and insomnia aged 13 to 17 (4 males) were included in the study (Table 1). The participating adolescents with ASD were recruited through information from Child and adolescent psychiatry in Stockholm. All participants fulfilled the general inclusion criteria for the pilot study: a prior community-based clinical consensus diagnosis of ASD (F84.5 or F84.9) according to ICD-10 (International Classification of Diseases World Health Organization [WHO], 1992) corroborated by the Autism Diagnostic Observation Schedule or the Autism Diagnostic Observation Schedule second edition (Lord et al., 2000); IQ >70 according to the Wechsler Intelligence Scale for Children fourth or fifth edition (Wechsler, 2004, Wechsler, 2016); insomnia according to the ICD-10 (G47.0 Insomnia), confirmed by an interview based on DSM-5 (American Psychiatric Association, 2013). Some participants had co-occurring diagnosis such as attention deficit disorder (F90.0), generalized anxiety disorder (F41.1), and obsessive-compulsive disorder (F42.2). Exclusion criteria were the presence of clinically assessed self-injury, high risk of suicidal behavior, antisocial personality disorder, borderline personality disorder or any form of schizophrenia or related disorders; substance abuse as well as physical illness or conditions that influence sleep (e.g., sleep apnea), the use of prescribed medications during the treatment for sleep problems and expressed lack of motivation to participate. The use of medication for other symtoms was not an exclusion criteria as long as the use was stable (type and dose) and was not prescribed for sleep problems. Three individuals who initially were included in the study did not start the treatment due to family related issues and concurrent daily activities.

**Table 1:** Participant characteristics.

Responder (R)	Age	Gender	ASD-diagnoses	Sleeping disorder diagnosis	Co-occurring diagnoses	Medica-tions	ISI pre-treament	AIS pre-treament	Parent
R1	14	M	F 84.5	G 47.0	F42.2		11	10	Mother
R2	16	M	F 84.9	G 47.0			13	11	
R3	17	M	F 84.5	G 47.0	F90.0C		16	14	Mother
R4	13	M	F 84.5	G 47.0			12	9	Mother
R5	16	F	F 84.5	G 47.0	F42.2		11	7	
R6	17	F	F 84.5	G 47.0	F90.0C, F41.1	Flouxetine	14	9	Mother

Note: Diagnosis according to ICD-10: F 84.5 = Asperger syndrome, F 84.9 = Pervasive developmental disorder, unspecified, G 47.0 = Insomnia, F90.0C = Attention Deficit Disorder (ADD), F41.1 = Generalized anxiety disorder, F42.2 = Obsessive Compulsive disorder.

### 2.3 Cognitive behavior therapy for insomnia

The internet-based iCBT-I was delivered through a platform, www.iterapi.se/sites/saga (Vlaescu et al., 2016). The treatment was based on a manualized, structured CBT for insomnia (CBT-I) (Sánchez-Ortuño and Edinger, 2015) and was delivered through psychoeducational videos. The treatment contained a combination of behavioral and cognitive interventions: sleep hygiene, sleep restriction therapy, stimulus control, focus on safety behavior, home assignment, problem-solving, sleep diary, cognitive restructuring, and applied relaxation. The treatment was adapted for adolescents with ASD by a supplementary manual for parents, anparent involvement. In further adapting the treatment to make it suitable for adolescents with ASD, particular attention was paid to language (vocabulary that was not to advanced) and examples that could be relevant to the group. The program was delivered through the Internet for 8 weeks, one module per week. See detailed information about the treatment content and structure in Table 2. A new module was available every Friday around lunchtime to allow the participant to look at the material during the weekend and for parents to support their children if needed. It was mentioned that participating would require about 1 h of work with the treatment per week, which was recommended to be spread out over different days.

**Table 2:** Treatment content and modules.

Module^a^	Structure	Content	Homework
1	Psychoeducation	*Introduction*, Sleep problems and CBT model.	
	Information about behavioral interventions	*Stimulus control*, behavioral treatment that aims to change behaviors associated with bed and bedroom and establish consistency in sleep patterns. Techniques include restricting bedroom for sleep only; going to bed only when sleepy; avoiding reading, television, phone, etc., in the bedroom; leaving the bedroom when unable to sleep; regular sleep schedule.	- Register the questionnaire: Stimulus control and change your sleep environment
		*Sleep restriction*, behavioral intervention that limits time in bed to sleep time, gradually increasing time in bed as sleep efficiency improves. Techniques include setting strict bedtime and rising schedules, and keeping a set wakeup time, with modifications based on sleep efficiency after a certain duration of time.	
		*Sleep hygiene*, bedroom circumstances and/or lifestyle factors perpetuating insomnia disorder, e.g. light exposure in bed and consuming caffeinated beverages in the hours before bedtime.	- Individualized procedures based on the need for better sleep emvironments.
		*Sleep diaries* were used to calculate a sleep schedule for the coming week, which was then reviewed the following week.	- Register sleep diaries during the week.
2	Psychoeducation	*CBT model, daytime*	- Register the questionnaire: Stimulus control - Individualized homework implementing sleep restrictions.
	Applied relaxation	*Progressive relaxation*, training to reduce somatic tension and control bedtime thought patterns that impair sleep. Techniques include progressive muscle relaxation, guided imagery, and paced breathing.	- Register sleep diaries during the week -Register your relaxation diary
3	Psychoeducation	*“Myths about sleep”* We go through the common notions of sleep, for example, that bad sleep does not affect health, that you only focus on the number of hours of sleep to have a good or bad sleep.	- Register the questionnaire: Stimulus control
	Cognitive interventions	*“Time for sleep-related or sleep-interfering worry and rumination” focuses on cognitive restructuring,* which is an intervention that aims to change how patients think about sleep by identifying, challenging negative thoughts, and replacing dysfunctional beliefs and attitudes. Dysfunctional beliefs create tension, impair sleep, and reinforce the beliefs. The intervention includes challenging notions about requisite amounts of sleep, notions that sleep is out of their control, and fears about missed sleep; thought journaling; and behavioral experiments around sleep beliefs.	- Register your negative thoughts and problem solving
	*Problem solving*	This component uses with the objective to improve problem solving skills, it is believed that worry (and sleep difficulties) will be reduced. This intervention involves psychoeducation about the importance of effective problem-solving.	- Register sleep diaries during the week
	Applied relaxation	*Short-term relaxation*, is used to reduce the level of tension, anxiety or stress. And to facilitate the participant falling asleep. The short relaxation is about 5–7 min (voluntary).	
4	Cognitive intervention	*Sensory impressions*, Attentional bias misperception of sleep and daytime deficits and monitoring for sleep-related threat.	- Register the questionnaire: Stimulus control - Register your negative thoughts and problem solving.
	Applied relaxation	*Conditional relaxation,* focuses on slowly tensing and then relaxing each muscle group.	- Register sleep diaries during the week.
5	Cognitive intervention	*Safety behavior,* the participant identifies the safety behaviors that maintain unhelpful beliefs by and they are encouraged them to challenge these.	- Register ABC
	Behavioral intervention	*Behavioral analysis* (antecedent, behavior, consequence, ABC)	- Register the questionnaire: Stimulus control
	Applied relaxation	*Differential relaxation* is a technique for exerting only the amount of muscular tension or energy required to perform an activity successful (voluntary)	- Register sleep diaries during the week
6	Psychoeducation	*The balance between activity and recovery* *exercise* and daylight	- Register ABC - Register the questionnaire: Stimulus control
	Applied relaxation	*Short-term relaxation (optional)* If the adolescent will continue use the relaxation technique they can continue with the short relaxation is about 5–7 min (voluntary).	- Register sleep diaries during the week
7	Behavioral intervention	*Power-nap,* is introduced to deal with fatigue (short term coping strategi) and at the end of the intervention. The objective is to increase compliance of treatment and the maintenance of the acquired skills after the treatment is concluded.	- Register your experiences and conclusion about the treatment Register sleep diaries during the week
	Applied relaxation	*Short-term relaxation (optional)* If the adolescent will continue use the relaxation technique they can continue with the short relaxation is about 5–7 min (voluntary).	
8	Maintaining Change	Together with the young people and the parent, the therapist goes through “Your tailor-made maintenance plan to encourage the adolescent to continue with the treatment and prevent relapse. Futhere the adolescent gives a evaluation about the treatment.	- Register your maintenance plan - Evaluation of the treatment

a The modules were weekly delivered.

There were two telephone support calls by a practitioner per week; 10 min on Monday to answer questions related to the module and 15 min every Friday to give support concerning the treatment. One participant received support by chat instead of telephone call by request. The telephone support was administered by three practitioners (female psychologists, 30–31 years; female CBT-psychotherapist, 45–47 years; male CBT-psychotherapist, 50–52 years) with experience in working with sleep disturbances and with adolescents with ASD. The practitioners followed a detailed treatment protocol for therapist and received a collegial supervision about the treatment fidelity.

### 2.4 Qualitative interviews

The participants were encouraged to share their experiences and perspectives using the qualitative general interview guide approach (the interviews were conducted by a female psychologist, 34–36 years of age who took no part in the treatment). The resulting verbal data were investigated using thematic analyses (Braun and Clarke, 2006). A general interview guide was developed to explore the participants' experiences and opinions in the current pilot study. The interview guide contained questions on how the participants experienced the structure and the content of the treatment as well as any perceived outcome of the treatment, such as changes in their sleeping patterns, whether they experienced any problems with undertaking the treatment, and what improvements they would recommend, enhancing the adaptation of this treatment for young people with ASD (see Appendix 1). The structured interview was conducted within two weeks after finishing treatment with all participants. Some participants completed the interview individually and some by the help of a parent, by their own request. The duration of the interviews was 29 to 51 min.

### 2.5 Quantitative measures

During the treatment, the participants completed the *Insomnia Severity Index* (ISI) and the *Athens Insomnia Scale* (AIS) self-rating measures at pre-, mid-, post-treatment and follow-up. The ISI (Bastien et al., 2001) is a seven-item measure that yields a quantitative index of sleep impairment and treatment outcomes. The Swedish version of the ISI has acceptable internal consistency (Cronbach's = 0.88) (Dragioti et al., 2015). The AIS (Soldatos et al., 2000) consists of eight items; the first five items assess difficulty with sleep induction, awakenings during the night, early morning awakening, and total sleep time and overall quality of sleep, while the last three items pertain to the next-day consequences of insomnia (problems with sense of well-being, overall functioning and sleepiness during the day). The validation of the internal consistency in a study showed a Cronbach's alpha of 0.90, the item correlation coefficient was about 0.70, and the test-retest reliability correlation coefficient was 0.90 at a 1-week interval (Soldatos et al., 2000). A *sleep diary* was used to calculate, in minutes per day, mean onset latency (SOL) and mean time spent awake during the night wake after sleep onset (WASO). The sleep diary also asked participants to indicate how rested they felt after waking with lower scores reflecting feeling more rested (FR), as well as their early morning awakening (EMA). We measured the total sleep time (TST) and the sleep efficiency (SE = total sleep time/allotted time in bed).

## 3 Data analysis

### 3.1 Qualitative analysis

The interviews were audio-recorded and transcribed verbatim and then coded from condense interview material into consistent emergent themes using thematic analysis (Braun and Clarke, 2006) aided by NVivo 11 (Edhlund and McDougall, 2016). Verbal material was double coded by two members of the research team. Thematic analysis was conducted as follows: (1) familiarization with the transcribed material, (2) generation of nodes by the independent rater through reviewing the verbal data using NVivo 11, (3) the generation of nodes by the second independent rater through review of the verbal data using NVivo 11, (4) identification of the themes by grouping of the initial nodes into themes (e.g. “improved sleep”), (5) similar possible themes were classified into larger groups; revalidation of themes against initial verbal data, (6) finalizing definition and labels of themes. No additional themes (only statements assignable to existing themes or subthemes) emerged after the analysis. The reliability and validity of the coding were considered satisfactorily stable and consistent at 79 to 100% uniformity. The agreement was 83%.

### 3.2 Quantitative analysis

Due to the small sample size in the current study, the preliminary efficacy of iCBT-I on the two self-report measures (AIS and ISI) and sleep diary parameters were investigated with within-group effect size estimations. Within-group effect sizes (Hedge's *g*) were calculated [(pretreatment minus post-treatment and post-treatment minus 6-month follow-up)/pooled and weighted standard deviation] with correction for small sample size to estimate the magnitude of improvement associated with iCBT-I. A three-fold classification of effect sizes has been suggested (Cohen, 2013): small (0.20–0.49), medium (0.50–0.79) and large (0.80 and above).

## 4 Results

### 4.1 Qualitative results

Thematic analysis of the interview data revealed five themes: experience of the structure of the treatment, treatment content, experienced outcomes, experienced difficulties, and suggested improvements. See the themes, subthemes, and examples in the Table 3.

**Table 3:** Results of thematic analysis.

Theme	Sub-theme	Example
*Structure of the treatment*	- *Treatment duration*	“Time is needed to create new habits” (Mother of R3)
	*- Treatment structure*	“The theoretical material was well adjusted for eight weeks” (R2); “It was easy to understand what to do” (R1)
	*- Time spent*	“…maybe half an hour… a day” (R4)
	*- Homework*	“It was boring but it had to be done so I did it” (R4), “It was good as it had a clear purpose” (R2)
	*- Telephone support*	“It was hard in the beginning since I had to talk” (R4). “She explained things very well” (R5)
*Treatment content*	*- Progressive muscle relaxation*	“It made me calm…“(R4), “it was good to use in everyday situations when you feel stressed and then you have a method to try to take control of the situation” (R2)
	*- Safety behaviors*	“I had to think about what behaviors were functional or not” (R2)
	*- Peace of mind*	“It was a way “to fall asleep” (R1), “… and gives you a “mental relaxation” which is quite nice (R1)
	*- Sleep diary*	Sleep diary was helpful to “become aware” (mother R4)
	*- Sleep hygiene*	“Yes, it just worked quite well for me because then it becomes a bit like this that I do not get distracted by things just because … because I had not really thought about that I was stressed out because my school things were kind of opposite my bed.” (R5) [authors clarification: it helped me get rid of things that cause stress and distractions in my bedroom which worked quite well for me]
	*- Sleep restriction*	“It was good… that I checked it. Because then I could write that time. I was up on average, send it to them and so they read it and they can give you…ehh… the results.” (R4) [authors clarification: it was good, it helped see average time and get feedback]
	*- Stimulus control*	“I thought it was good that you did not get stuck in the bed too long when you could not sleep but varied it with other things and then it was quite often that you could then fall asleep easier” (Mother R4) [authors clarification: it helped to leave the bed when it was hard to fall asleep, quite often he fell asleep easier afterwards]
	*- Problem solving*	A way to cleanse your thoughts… to get rid of everything that is stressful.” (R5)
	- General experience of the treatment	“In general, I think I'm very happy with the treatment and so, it's good that it exists and it's very smooth, especially in these corona times to run it digitally.” (Mother of R1) “…you are supposed to learn how to live with this, a way to act in the future… all your life really.” (R3) “I think that what was good with the treatment was that there was a program you had to follow every week that would eventually lead to you having the complete package with different strategies and possible solutions.” (R2)
Experienced outcomes	-Insights	“There is another awareness on how all my actions influence my sleep.” (R5) “it was good information, a lot that seemed obvious but you noticed that whops did we end up here”[authors clarification: it was good, a lot seemed obvious but we noticed how our actions had brought as to this point] (Mother of R3)
	- Cognitive and behavioral changes	“…the exercises helped to structure the sleeping pattern and to understand what caused [safety behavior] me to sleep poorly… it made me think about my sleep and gave me different strategies regarding how to treat it.” (R2) “There is another awareness of how all my actions influence my sleep… now I don't have to think as much about how I sleep and about sleep in general. It is not something that consumes my entire day” (R5). “You improve your ability to concentrate… that is the biggest difference I notice when I sleep better.” (R2)
	- Specific changes in relation to sleep	“I sleep better”, “I sleep more hours now, after the treatment”, “my feeling is that it takes less time to fall asleep.” (R1)
	- Adverse events	“When I have been lying in bed for a long time… then I feel some kind of guilt that I didn't feel before guilty, that now I know it's you know not good” (R5) [authors clarification: when I do things I have learned are not good for my sleep I feel guilty] “at the end of the treatment there was a bit too much… it felt overwhelming” (Mother of R1)
Experienced difficulties	*- Parental support*	“We divided it a bit… depending on the energy level after school…we added it to the schedule” (Mother of R4) “we have it together all the way, otherwise I think it would have been hard for X to take the initiative, to remember it, to take the initiative to do it and actually, do it, but with my support it went well.” (Mother of R1)
	*- Flexibility*	“Some things were very hard… mostly the times” (R3) [authors clarification: it was hard to change things needed, especially in regards to time in bed]
	*- Initiation*	“It was hard at first.” (R2) “it depended on, if I had work that week, and if I was a bit tired, then it really made me get started on the module that came out on Friday, on Sunday, cause I postponed and postponed… but sometimes it was kind of like I was like, I was really eager and looked at it when I came home from school on Friday, or almost looked at it on the bus on the way home from school” (R5)”[authors clarification: if I had work or was tired, I postponed getting started, but sometimes I was really eager and started as soon as I could”]
	*- Attention deficits*	“I just listened to 5 min and then I said no, it's too long.” (R4) “It was hard to get started.” (R5)
	*- Planning*	“It didn't go so good… the calls [telephone support]. I feel like it had to be done by that time” (R5)
*Suggested improvements*	*- Specific proposals for change*	“That it was written down somewhere when the like, kind of, when the call was planned” (R5) “the applied relaxation was very long” (R6)
	*- Technical improvements*	“I did not have a text version of the videos and… that you could read while listening.” (R5) “so instead of having to log in every time so we could listen to it” (Mother of R4)” [authors clarification: the possibility to download files to a mobile phone”]
	*- Content that needs to be clarified*	In regards to sleeping restriction: “it was hard to understand at first” (R5)

#### 4.1.1 Structure of treatment

The theme consisted of five specific subthemes: treatment duration, treatment structure, time spent, homework, and telephone support.

All participants (responders [R]) reported that the treatment duration of eight weeks was good, and some participants expressed their preference for a longer treatment duration. The participants reported satisfaction and positive experiences regarding treatment structure. They found it easy to follow, limiting mental energy required since the structure was the same and predictable e.g., the disposition using the weekend to go through the new module which was delivered at the end of a week. The time spent on the treatment ranged from 30 to 45 min/week to 1 h/day, but the time spent depended on which part of the treatment the participant was going through. Most of the participants reported that homework was demanding or boring. On other hand, it was reported as helpful in solving problems and useful. The telephone support (one had chat-based support) was helpful according to the participants.

#### 4.1.2 Treatment content

The theme consisted of eight subthemes: progressive muscle relaxation, safety behaviors, peace of mind, sleep hygiene, sleep diary, sleep restriction, stimulus control, and problem-solving. The progressive relaxation, particularly the shorter version, was appreciated by the participants as it had a positive effect on their well-being, such as helping with anxiety and stress. The longer version of applied relaxation was perceived as too long, and few used the intervention. Identification and managing safety behaviors and peace of mind were helpful according to most of the participants. Some participants reported that catastrophic thoughts hindered the effect of the exercise.

Some of the participants (three) reported positive experience with sleep restriction, three mentioned difficulties understanding the intervention/instructions and two participants reported never using the intervention. Two participants reported that the sleep diary was helpful, e.g., to “become aware” (mother of R4) of their own sleep patterns and perceived it as positive “recurring points” (R5) in the treatment. Four participants reported making several changes to their sleeping environments and they reported this having a substantial effect. In addition, they experienced a good effect of stimulus control. Four participants reported problem-solving as having a good effect. The theme general experience of the treatment shows that all participants had an overall positive experience of the treatment. Some of the participants stated that the treatment was “good in its entirety and I understood the content well” and that it gave hope. Two mothers stated being very pleased with the treatment and with the fact that it was offered online. The treatment was also experienced as being informative and something to apply and return to in the future. One of the participants reported that the treatment provided a new way of thinking about their sleep problems, new behavior leading to a reduction of the symptoms. Despite these positive experiences, one participant reported negative experience such as feeling stressed due to lack of time to complete the homework or implementing the long version of applied relaxation.

#### 4.1.3 Experienced outcomes

The theme consisted of four specific subthemes: *insights*, *cognitive and behavioral changes*, *specific changes in relation to sleep*, and *adverse events*. The participants (four) mentioned *insights* and better understanding of the nature of their sleep and what affected their sleeping patterns reducing safety behaviors and aiding better sleep, e.g., one participant increased the number of nights sleeping in their own bedroom by 79%. All participants experienced positive *changes in behavior* and/or *cognitive* patterns such as awareness of how a stressful day impacts their sleep pattern and how to manage this as well as the ability to focus on the small improvements. In addition, it increased awareness about their sleep problems among their family members.

The participants experienced specific changes after the treatment, such as being more calm/relaxed/in a better mood. Two participants stated that there was less worry/anxiety at night, and a reduction in sleep-related catastrophic thoughts, which was a problem prior to the treatment. Some stated that the iCBT-I helped to reduce stress. In summary, most of the participants reported iCBT-I as having a good outcome, reporting better sleep after completing the treatment. One participant reported fluctuating problems with sleeping and could therefore not yet assess any potential outcome at the time of the interview.

Regarding *adverse events* of the treatment, a mother reported having to quit her own studies to support her son. One participant reported “feeling guilty” (R5) as an effect of increased awareness when doing something that could have a negative effect concerning sleep. The same participant (R5) also reported that waiting to become sleepy, as recommended in the treatment, could lead to having very few hours of sleep due to sleep restriction.

#### 4.1.4 Experienced difficulties in completing iCBT-I

The theme consisted of six specific subthemes: *parental support*, *flexibility*, *initiation*, *attention deficits*, and *planning*. Half of the participants received extensive *parental support* to implement the treatment. The parents (mothers) planned and organized all that was needed, e.g., adding the treatment to the participant's daily schedule, and they all helped the participants to get started with the modules and homework. They reported also helping them to complete the tasks, such as looking at the modules together. According to some (three), participants, the *telephone support* was perceived as a checkpoint that prompted the participant to do the homework. In addition, the treatment was described as demanding as it was hard to change things to some degree, *relating to cognitive and behavioral flexibility*, and it was hard to find time for the telephone call which led to their “routine being disturbed” and for the homework. Another participant found it hard to change specific things like rearranging the bedroom and trying to implement problem-solving earlier during the day, and one reported difficulties with initial changes but that it was easy once that participant started the intervention.

Apart from the three participants that needed extensive parental support, one other participant reported difficulties getting started (procrastination) and difficulties due to lack of planning/organizing, and one reported difficulty staying focused. None of the participants completed all components and homework, according to their own verbal reports. This was described as being related to lack of time, lack of motivation for a particular intervention or problems understanding the task or how to execute it.

#### 4.1.5 Suggested improvements of iCBT-I

The theme consisted of three specific subthemes: *specific proposals for change*, *technical improvements*, and *content that needs to be clarified*. Specific proposals for change were addressed, such as chat support instead of telephone support and the need for a predictable time for telephone support each week, which preferable could be registered in writing on the treatment homepage. Further, more support for treatment planning was mentioned, such as a schedule that could work as an example of how to plan for exercises and time management. A parent proposed more intuitive labelling for different exercises to aid a faster understanding of the content when approaching the module/exercise. Regarding technical improvements, the participants suggested a written version of the videoclips in the modules, information about the length of the videos in the modules, and that the structure of the webpage could be easier to get acquainted with and the possibility of saving the exercises on the phone instead of needing to log on to the webpage to get access to the files, to make them more easily accessible. The participants' views on the clarity of the treatment content revealed the need for more clarification regarding primarily “sleep restriction”, “sleep diary” and “safety behaviors”.

### 4.2 Quantitative results

#### 4.2.1 Self-report measures: from pre-treatment to 6-month follow-up

The means, standard deviations, and within-group effect sizes for the AIS and the ISI from pre-treatment to 6-month follow-up are displayed in Appendix 2. From pre- to post-treatment, the effect sizes for the AIS and the ISI total scores were large (*d* = 1.85–2.30). Based on the within-group effect sizes, there were small improvements from post-treatment to 6-month follow-up on the AIS and the ISI (*d* = 0.22–0.38).

### 4.3 Sleep diary outcomes: from pre- to post-treatment

In Appendix 2, the means, standard deviations, and within-group effect sizes for the sleep diary outcomes from pre- to post-treatment can be seen. From pre- to post-treatment, the effect sizes were large for four of the five sleep diary parameters (SOL, WASO, TST, and SE). On one of the sleep diary variables, EMA, there was a moderate deterioration from pre- to post-treatment; note, however, that the average pretreatment EMA score was very low and increased to a still low value at post-treatment, see Appendix 2.

## 5 Discussion

The objective of this pilot study was to investigate the experiences of adolescents with ASD and sleep problems about iCBT-I after completing the treatment and the feasibility of the treatment. A mixed-method approach that involved both a qualitative and quantitative evaluation was applied. Thematic analysis was used for the qualitative investigation. All participants reported a positive experience of the treatment, indicating that iCBT-I is a feasible treatment choice for this group. The results suggest that iCBT-I was helpful in achieving better sleep. Our quantitative analysis confirmed the results of the thematic analysis. Based on effect size estimations on two self-report measures (AIS and ISI) and five sleep diary parameters, iCBT-I showed a large, preliminary effectiveness.

The participants reported insights into their own sleep patterns, behavioral and cognitive changes, and positive sleep-related changes. The iCBT-I was described as informative and helpful, supporting earlier findings (McCrae et al., 2020a, McCrae et al., 2020b). The majority of the participants reported the treatment aiding their understanding of the relationship between sleep and affecting factors in their everyday life, and that it gave hope and specific strategies until the participant had “the complete package” (R2). All participants experienced changes in terms of sleeping, such as increasing sleep duration, falling asleep faster, or generally sleeping better. In addition, they experienced changes in behavior and/or cognitive patterns as a positive outcome of the treatment, such as overall improvement in psychological health (calmer/better mood) and less time spent worrying or thinking about sleep in a stressful way. The structure and content of the treatment were perceived as positive by the participants. Some interventions, such as the longer version of the relaxation, was not used as intended as it was perceived to be too long, and half of the participants reported *safety behaviors* as helpful. Changes in the sleeping environment were reported as efficient. The structure of the modules was experienced as positive (e.g., the structure being seen as predictable) or neutral. The predictability of everyday activities seems to be helpful for individuals with ASD due to a heightened intolerance of uncertainty, which is closely associated with anxiety and sensory sensitivities in ASD (Neil et al., 2016).

In addition, some needs for treatment improvement were identified, such as support for the custodians of some participants, support to enhance participants' cognitive and behavioral flexibility, and the need for support in planning/organizing the implementation of the treatment and homework. In addition, some participants described the tendency of feeling guilt and increased worrying due to increased awareness of sleep problems. Some participants experienced stress as a result of concurrent activity, such as having a lot of schoolwork. None of these difficulties were of such severity that they were considered to have an impact on treatment outcome. However, these reports indicate the need for improvements of the treatment, adding knowledge of the heterogeneity of the ASD population and the need for individual adaptations, especially in telephone support by the practitioners. In terms of treatment duration, the iCBT-I was delivered in eight weeks. Some participants had access to the treatment for more than eight weeks after completed the follow up data collection. In addition, technical improvements to facilitate the treatment experience were also suggested, such as written information from the modules about the content and duration of the videos.

There are several limitations that need to be addressed. First, the sample size was small, limiting the generalizability of the results. However, the results of our study show the usefulness of qualitative designs in addition to quantitative evaluations. Second, the difficulty in communication and abilities in verbal expressiveness in individuals with ASD (Losh and Gordon, 2014) limits the possibility of generating comprehensive verbal data to answer the research questions. However, the fact that custodians participated in four of the interviews, and actively in three of these, might have compensated for individual verbal limitations in these cases. Third, this study only included those who completed the treatment, excluding individuals (n = 3) who did not start the treatment. Thus, the finding might be overly positive, and negative experiences as well as added views on further adaptations needed might have been lost. Fourth, the majority of the participants of the study had co-occurring diagnosis adding to the complexity of the study, which can be argued to be a strength of the study as this is the rule rather than the exception in clinical reality (Simonoff et al., 2008; Mukaddes et al., 2010; Matson and Williams, 2014), and it might affect treatment outcomes (Lerner et al., 2012) and the experience of the treatment. Fifth, the collected data were subjective in nature, which limits the generalization of the results, but on the other hand, they offer individual experiences that are important to understand things in more detail. The study adds several new aspects that may lead to further research in this field. Sixth, lack of detailed description of the parent involvement in the treatment may limit the generalizability. A study shows that parents´ attitudes and beliefs about their autistic children or adolescents may be of importance in their child sleep treatment. A study showed that parents of children and adolescents with ASD tend to believe that sleep problems of their children and adolescents with ASD are more intrinsic, less amenable to change, and less responsive to treatment compared to typical developed children, but they still moderately agree that their children may be receptible to change and can be helped with treatment (Bessey et al., 2013). On the other hand, the parental involvement in treatments may be more advantageous in adolescents with ASD (Adam et al., 2007; Conroy et al., 2015). The parent involvement could lead to the maintenance of the acquired behavior change (Malow et al., 2006; van Deurs et al., 2019). In regard to parent involvement in some interviews in the qualitative part of the study, it gave additional perspectives about the strengths and potential improvements of the treatment before implementing an efficacy study with a large sample size.

The strength of the study is that it is a first feasibility study of iCBT-I in adolescents with ASD and their perspectives on this treatment, which has the immediate effect that the research team can further adapt the treatment before offering it as a part of a large-scale randomized controlled trial. The study has found important factors to consider in future treatments for this group.

## 6 Conclusions

There is a lack of sufficiently evidence-based intervention methods for adolescents with ASD and insomnia. The present pilot study is the first to investigate participants' experiences and views of the iCBT-I adapted to adolescents with ASD. Our results yielded important information about potential improvements of iCBT-I prior to starting an efficacy study. Feedback from participants and parents provides valuable insights for further improvements of internet-delivered CBT for this population.

Future studies should focus on the efficacy of the adapted iCBT for adolescents with ASD and insomnia and tailor the treatment according to their specific needs for support. Further cognitive aids regarding treatment should be considered. Further development of treatment structure may enhance treatment adherence. Future studies can also be recommended to include a broader spectrum of outcome measures such as parental factors and the overall life satisfaction of the participant.

## Formatting of funding sources

Funding: no funding was received for this study.

## Ethical approval

Approval for all study procedures was obtained from the local ethics committee in Stockholm, Sweden (2017/1399-31).

## Consent to participate

Informed consent was obtained from all individual participants included in the study.

## CRediT authorship contribution statement

All authors contributed to the study conception and design. Material preparation, data collection and analysis were performed by Nora Choque Olsson, Markus Jansson-Fröjmark, Lisa Georén, Gerhard Andersson, and Lisa Nordenstam. The first draft of the manuscript was written by Lisa Georén, Nora Choque Olsson, Markus Jansson-Fröjmark and Gerhard Andersson. All authors commented on previous versions of the manuscript. All authors read and approved the final manuscript.

## Declaration of competing interest

The authors have no relevant financial or non-financial interest to disclose.

## Figures and Tables

**Table:** 

Topic	Examples of guide
Introduction	Hi. My name is … and I am interested in how the CBT treatment for sleep problems on the internet went. I want to know more about how participants experienced the treatment and if it has changed something for them… We want to hear both things that you have experienced as good and bad… everything you say will be confidential
Structure and repetition of treatment	I know that every week you usually go through new information and exercises.… Do you remember what you learned? [Give examples if necessary] In this treatment, each new module was laid out in much the same way. What did you think of that? How did you experience the telephone support?
Content	Now I'm interested in what you thought about the different parts of the treatment. What did you think about….? [Explain and give examples of each point if necessary.] Was there anything you didn't understand in the treatment? What part of the treatment do you think worked best for you?
Treatment outcome	Is there anything you think has become better after the treatment? Is there anything that has become worse? Besides this, have you experienced any other effects?
Any problems in implementing	How difficult did you find it to get started with the module and doing the homework? Did you find it difficult to change things? [Give examples of changes that were required]
Final words on the treatment	When you look back at the treatment, how do you think it was?
End of interview	Thank you for telling us about how you experienced the treatment! Do you have any questions or concerns that you would like to share? Thank you for your time!

**Table:** 

Measures	Pre [M (SD)]	Mid [M (SD)]	Post [M (SD)]	3-m follow up [M (SD)]	6-m follow up [M (SD)]	*d*: pre to post	*d*: post to 6-m
Athens Insomnia Scale (AIS)	9.3 (2.5)	7.2 (1.7)	4.4 (2.8)	4.3 (1.6)	3.8 (2.7)	1.71	0.20
Insomnia Severity Index (ISI)	11.8 (2.2)	9.5 (1.5)	6.4 (2.5)	6.3 (1.3)	5.5 (2.2)	2.12	0.35
Sleep onset latency (min)	64.6 (47.9)	46.6 (30.9)	22.7 (46.9)	–	–	0.82	–
Wake after sleep onset (min)	9.5 (6.5)	6.2 (3.9)	1.9 (5.1)	–	–	1.21	–
Early morning awakening (min)	1.2 (13.5)	3.7 (8.7)	8.9 (13.2)	–	–	−0.53	–
Total sleep time (hh:mm)	7:24 (0:42)	7:42 (0:27)	8:03 (0:41)	–	–	1.76	–
Sleep efficiency (%)	81.9 (6.5)	85.4 (4.2)	90.1 (6.4)	–	–	1.17	–

Abbreviations: ISI = Insomnia Severity Index; AIS = Athens Insomnia Scale. Follow-data on Sleep diary was not collected.
